# Supplementary material for: Acceleration-Dependent Effects of Vibrotactile Gamma Stimulation on Cognitive Recovery and Cholinergic Function in a Scopolamine-Induced Neurotoxicity Mouse Model
Source: Biomedicines. 2025 Aug 20;13(8):2031. doi: 10.3390/biomedicines13082031 (PMC12383785; doi:10.3390/biomedicines13082031)
Supplement: Supplementary file 1 [file biomedicines-13-02031-s001.zip › biomedicines-3787303-supplementary.pdf]

Supplementary Materials

Supplementary Methods

SH-SY5Y cells were differentiated in high-glucose DMEM supplemented with 5% FBS and 5 µM retinoic acid (RA) for 6 days. After differentiation, cells were exposed to 40 Hz vibrotactile stimulation at three different acceleration levels (1.6, 2.2, and 5.9 m/s^2) for 8 hours per day for 3 consecutive days. mRNA expression levels of GAP-43, DCX, NeuroD1, and MAP2 were quantified by RT-qPCR. All data are presented as mean ± SEM. The 2.2 m/s^2 condition yielded the highest expression across all markers and was selected as the optimal stimulation intensity for in vivo studies. RT-qPCR was performed using the following primer sets: GAP43 – Forward: 5′-AGA GGA ACC TGA GGC TGA CC-3′, Reverse: 5′-GCT AGT GGG TGG GAA AGG AC-3′; DCX – Forward: 5′-TAT GCG CCG AAG CAA GTC TCC A-3′, Reverse: 5′-CAT CCA AGG ACA GAG GCA GGT A-3′; NeuroD1 – Forward: 5′-GGT GCC TTG CTA TTC TAA GAC GC-3′, Reverse: 5′-GCA AAG CGT CTG AAC GAA GGA G-3′; MAP2 – Forward: 5′-TCA GAG GCA ATG ACC TTA CC-3′, Reverse: 5′-GTG GTA GGC TCT TGG TCT TT-3′; Each experimental condition was analyzed in three independent biological replicates, and within each biological replicate, three technical replicates were performed. For data analysis, the mean value of the three technical replicates was calculated for each biological replicate. These averaged values were then used to compute the mean ± standard error of the mean (SEM) across the three biological replicates, and individual data points in the figures represent each biological replicate's averaged value (Figure S2).

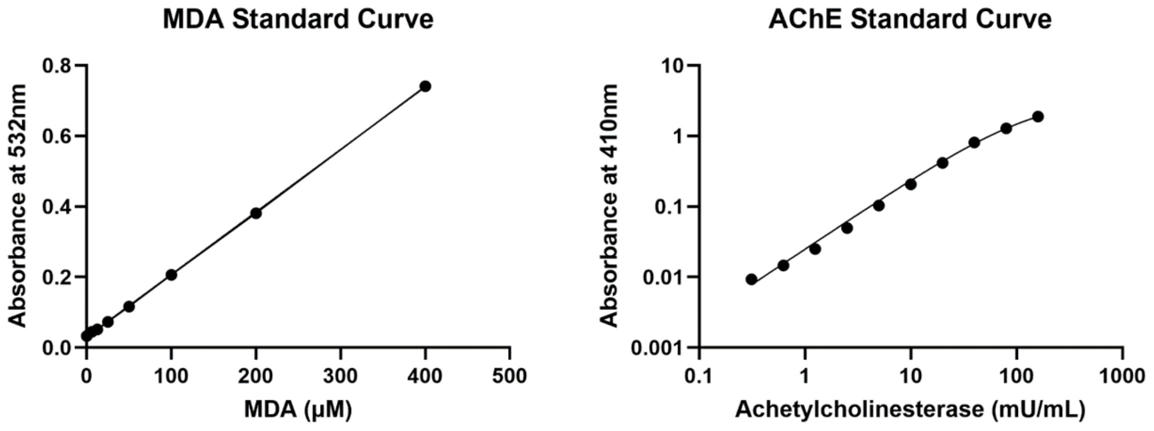

Figure S1. Standard curves for MDA and AChE assays. (A) Standard curve for malondialdehyde (MDA) quantification measured at 532 nm. Serial dilutions ranging from 0 to 400 µM were used to construct a linear regression model. Linear regression equation was y

= 0.0018x + 0.0303 with $R^2 = 0.9999$. (B) Standard curve for acetylcholinesterase (AChE) activity measured at 410 nm. A semi-logarithmic scale was used, with serial dilutions of AChE ranging from 0.1 to 500 mU/mL. These curves were used to calculate experimental sample concentrations. Regression equation was $y = 0.0239 \times x^{0.9054}$ with $R^2 = 0.995$

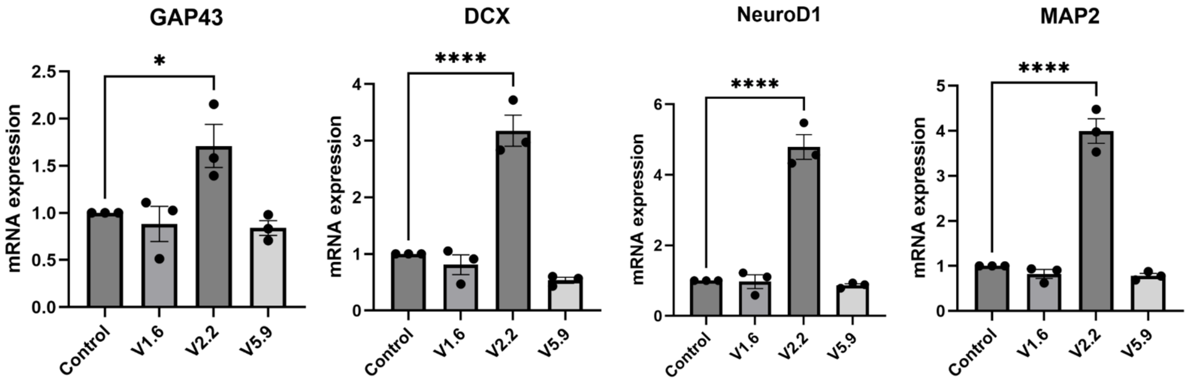

Figure S2. Optimization of vibrotactile acceleration for neurite marker expression in SH-SY5Y cells. Statistical significance is indicated as follows: *$p < 0.05$, ****$p < 0.0001$. All statistical comparisons were performed using one-way ANOVA followed by Tukey's multiple comparisons test. As control group values were normalized to 1, all biological replicates showed identical values, and therefore no error bars are visible for this group

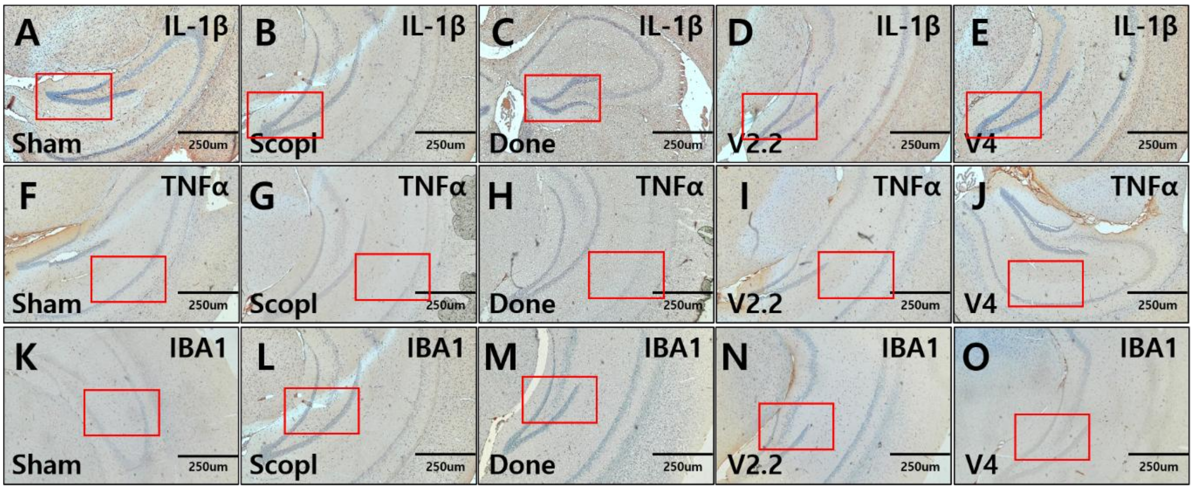

Figure S3. Overview of hippocampal immunostaining for inflammatory markers. Representative immunohistochemical images of whole hippocampal region stained for IL-1β (A–E), TNF-α (F–J), and IBA1 (K–O) at 40× magnification. Scale bars = 250 µm

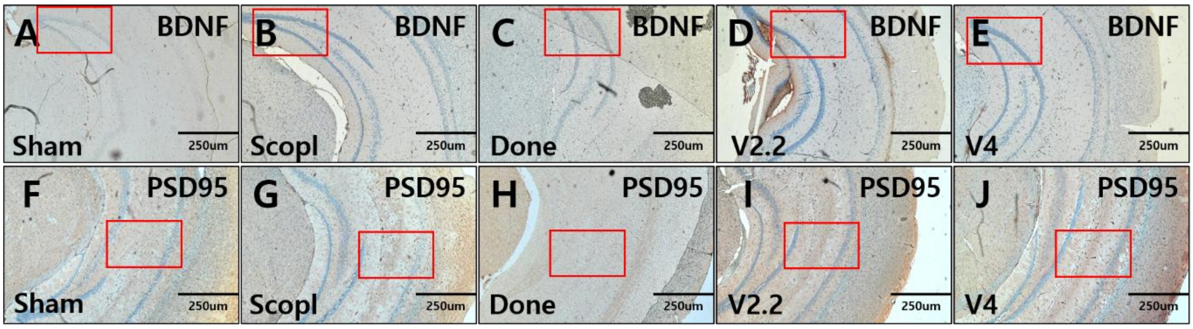

Figure S4. Overview of hippocampal immunostaining for neuronal markers. Representative immunohistochemical images of whole hippocampal region stained for BDNF (A–E) and PSD95 (F–J) at 40× magnification. Scale bars = 250 μm.

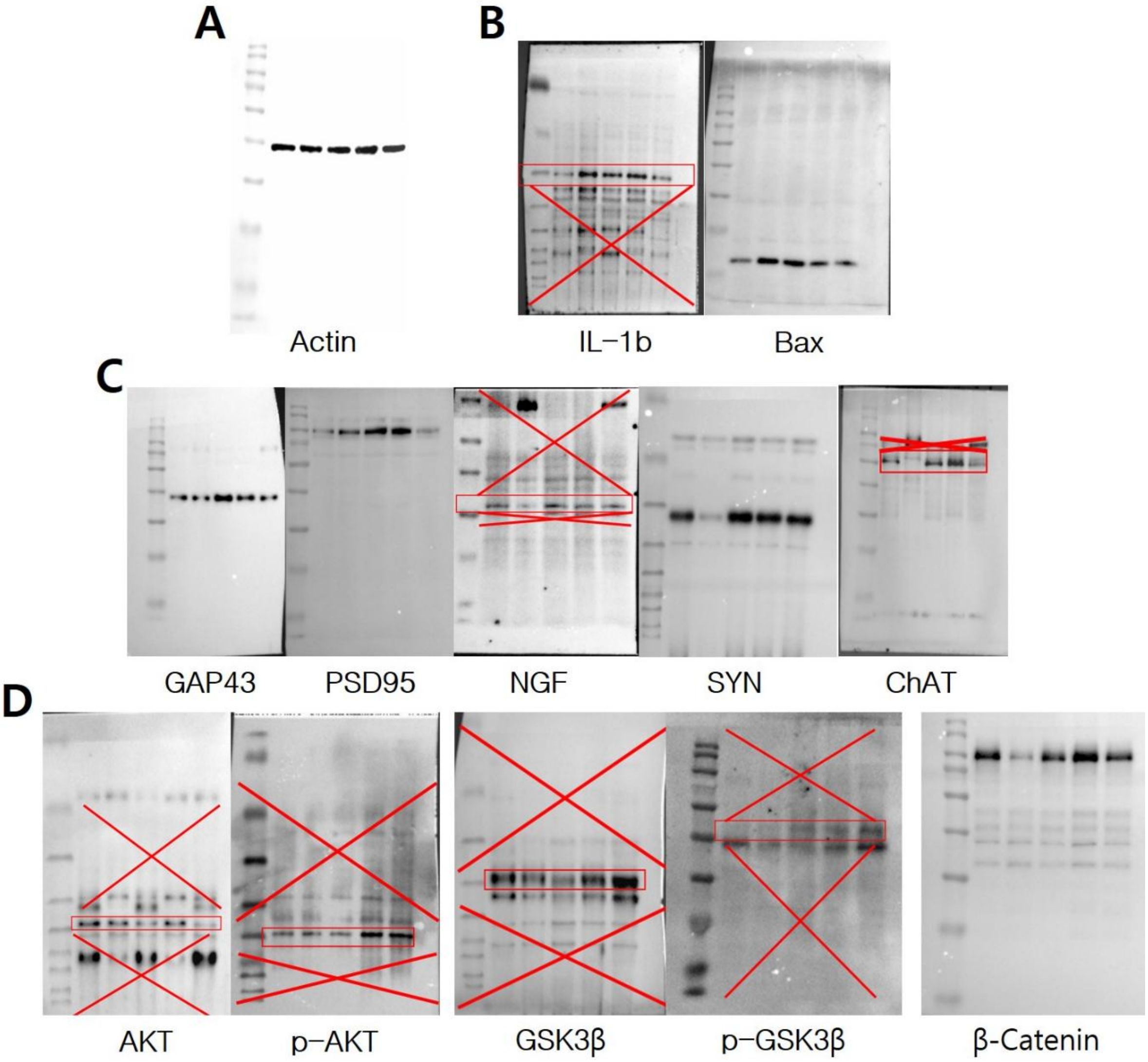

Figure S5. Full-length, uncropped Western blot images. (A) β-actin loading control obtained from identical protein extracts loaded in equal amounts for all experiments. (B–D) Target proteins probed on separate gels/membranes using same set of samples. Red boxes indicate regions used in main figures, and red crosses mark unused areas of the membrane. Molecular

weight markers are visible on the left of each blot.

Table S1. Humane endpoint criteria based on ethical considerations. This table outlines the humane endpoint criteria used in this study to ensure ethical treatment of animals. Animals were euthanized using CO_2 when scoring thresholds or clinical symptoms indicating significant distress or suffering were met. Only animals not meeting any humane endpoint criteria were included in the experiments.

Humane Endpoint Criteria from an Ethical Perspective

In this study using an inflammatory brain model, animals were euthanized using CO_2 under the following conditions: (1) a clinical scoring of 3 points for body weight change, 2 points for physical condition, or 3 points or more for behavioral change; or (2) the presence of corresponding clinical symptoms as described above. All experiments were conducted using animals that did not exhibit any of these humane endpoint criteria.

1) Scoring Assessment

a. Body Weight Change

Score	Body Weight Change
0	Normal
1	Less than 10% weight loss
2	10–15% weight loss
3	More than 20% weight loss

b. Physical Condition Changes

Score	Fur / Grooming	Eyes / Nose
0	Normal, well-groomed	Normal
1	Rough fur	Eyes partially closed or squinting, no tearing or nasal discharge
2	Rough fur, hair loss, ungroomed appearance	Eyes partially closed or squinting, tearing or nasal discharge, porphyrin stain

c. Behavioral Changes

Score	Posture	Activity
0	Normal	Normal
1	Sitting in a hunched posture	Reduced activity, moves after light stimulation
2	Hunched posture with head resting on the cage floor	No spontaneous activity, reduced alertness, moves only after moderate stimulation
3	Lying flat on the cage floor	Self-injury, severe hypoactivity or hyperactivity, no response to moderate stimulation

2) Clinical Symptoms

- **Tumor Growth or Related Effects**
  - · Tumor size exceeding 10% of the animal's normal body weight
  - · Tumor volume reaching 1 cm^3 on the skin

· Necrosis, infection, or ulceration interfering with feeding or drinking

- **Severe Organ or Systemic Symptoms**
  · Respiratory: labored breathing, coughing, rales
  · Cardiovascular: signs of shock, bleeding, hypersensitivity
  · Gastrointestinal: severe diarrhea or vomiting
- **Moribund State (Imminent Death)**

Table S2. List of primary and secondary antibodies used for Western blot analysis. Table includes antibody names, dilution ratios, sources (companies), and catalog numbers.

Antibody	Dilution	Company	Catalog Number
anti-β-actin	1:10000	Genetex	#GT5512
anti-IL-1β	1:1000	Genetex	#GTX74034
anti-BAX	1:1000	Genetex	#GTX109683
anti-GAP43	1:1000	Cell Signaling	#8945S
anti-PSD95	1:1000	Genetex	#GTX133091
anti-NGF	1:1000	Genetex	#GTX03258
anti-synaptophysin	1:1000	ABclonal	#A6344
anti-ChAT	1:2000	ABclonal	#A19031
anti-phospho-Akt(Ser473)	1:2000	Cell Signaling	#9271
anti-AKT	1:1000	Cell Signaling	#9272
anti-GSK3β	1:1000	Cell Signaling	#9332
anti-p-GSK3β	1:1000	Cell Signaling	#9322
anti-β-catenin	1:4000	Abcam	ab22656
Goat anti-mouse IgG HRP	1:3000	Abcam	ab6728
Goat anti-rabbit IgG HRP	1:3000	Abcam	ab6721

Table S3. List of primary and secondary antibodies used for immunohistochemistry. Table includes antibody names, dilution ratios, sources (companies), and catalog numbers.

Antibody	Dilution	Company	Catalog Number
anti-β-amyloid	1:400	Invitrogen	#44-344
anti-BAX	1:200	Genetex	#GTX109683
anti-AChE	1:200	Genetex	#GTX636298
anti-IL-1β	1:200	Genetex	#GTX74034
anti-TNF-α	1:200	Genetex	#GTX110520
anti-IBA1	1:400	Genetex	#GTX635363
anti-BDNF	1:100	Genetex	#GTX132621
anti-PSD95	1:1000	Genetex	#GTX133091
anti-rabbit/mouse HRP	-	Agilent	#K5007

Table S4. Post hoc comparison of behavioral test results between experimental groups. This

table presents pairwise *p*-values obtained from Tukey's multiple comparison tests for each behavioral assay: Morris water maze (MWM; day 4 latency), novel object recognition (NOR), and Y-maze spontaneous alternation test.

**MWM day4**	Sham	Scopolamine	Donepezil	V2.2	V4
Sham		0.0203	0.6104	>0.9999	>0.9999
Scopolamine			0.5406	0.0148	0.0086
Donepezil				0.6224	0.5019
V2.2					>0.9999

**NOR test**	Sham	Scopolamine	Donepezil	V2.2	V4
Sham		0.0452	>0.9999	0.857	>0.9999
Scopolamine			0.0693	0.0006	0.0312
Donepezil				0.6078	>0.9999
V2.2					0.7469

**Y-maze test**	Sham	Scopolamine	Donepezil	V2.2	V4
Sham		0.0003	0.0045	0.0017	0.0029
Scopolamine			0.7833	0.8846	0.7943
Donepezil				0.9989	>0.9999
V2.2					0.9995

Table S5. Post hoc comparison of results between experimental groups. This table presents pairwise *p*-values obtained from Tukey's multiple comparison tests for markers assessed by Western blot analysis and biochemical analysis.

**MDA**	Sham	Scopolamine	Donepezil	V2.2	V4
Sham		0.5758	<0.0001	0.0003	0.1662
Scopolamine			<0.0001	<0.0001	0.8904

Donepezil				0.9527	<0.0001
V2.2					<0.0001

**AChE**	Sham	Scopolamine	Donepezil	V2.2	V4
Sham		0.0016	0.1576	0.9059	0.8521
Scopolamine			0.1574	0.0084	0.0109
Donepezil				0.5319	0.6114
V2.2					>0.9999

**p-AKT**	Sham	Scopolamine	Donepezil	V2.2	V4
Sham		0.986	0.3971	0.0005	<0.0001
Scopolamine			0.2065	0.0003	<0.0001
Donepezil				0.0064	<0.0001
V2.2					<0.0001

**p-GSKβ**	Sham	Scopolamine	Donepezil	V2.2	V4
Sham		0.0003	<0.0001	<0.0001	<0.0001
Scopolamine			<0.0001	<0.0001	<0.0001
Donepezil				<0.0001	<0.0001
V2.2					0.9931

**β-catenin**	Sham	Scopolamine	Donepezil	V2.2	V4
Sham		0.0015	0.9993	0.9997	0.6993
Scopolamine			0.0011	0.0019	0.0092

Donepezil				0.9929	0.5702
V2.2					0.7966

**GAP43**	Sham	Scopolamine	Donepezil	V2.2	V4
Sham		0.8516	<0.0001	0.004	0.6798
Scopolamine			<0.0001	0.0178	0.9968
Donepezil				0.0008	<0.0001
V2.2					0.0298

**PSD95**	Sham	Scopolamine	Donepezil	V2.2	V4
Sham		<0.0001	<0.0001	<0.0001	0.0608
Scopolamine			0.0004	<0.0001	0.0001
Donepezil				0.0268	<0.0001
V2.2					<0.0001

**NGF**	Sham	Scopolamine	Donepezil	V2.2	V4
Sham		0.0064	0.1854	0.649	0.9467
Scopolamine			0.0003	0.0531	0.0023
Donepezil				0.0223	0.4728
V2.2					0.2883

**SYN**	Sham	Scopolamine	Donepezil	V2.2	V4
Sham		0.0133	0.002	0.8699	0.0813
Scopolamine			<0.0001	0.0033	0.0002

Donepezil				0.0078	0.1707
V2.2					0.3215

**ChAT**	Sham	Scopolamine	Donepezil	V2.2	V4
Sham		0.0423	<0.0001	0.0003	0.2766
Scopolamine			<0.0001	<0.0001	0.0018
Donepezil				0.6677	0.0007
V2.2					0.0044

**IL-1β**	Sham	Scopolamine	Donepezil	V2.2	V4
Sham		<0.0001	0.0004	<0.0001	0.007
Scopolamine			0.0005	0.0862	<0.0001
Donepezil				0.0336	0.25
V2.2					0.0013

**Bax**	Sham	Scopolamine	Donepezil	V2.2	V4
Sham		0.0002	0.0156	0.9996	0.2564
Scopolamine			0.0479	0.0001	0.003
Donepezil				0.0116	0.3988
V2.2					0.1961
